# Aluminum Alloy Anode with Various Iron Content Influencing the Performance of Aluminum-Ion Batteries

**DOI:** 10.3390/ma16030933

**Published:** 2023-01-18

**Authors:** Ghadir Razaz, Shahrzad Arshadirastabi, Nicklas Blomquist, Jonas Örtegren, Torbjörn Carlberg, Magnus Hummelgård, Håkan Olin

**Affiliations:** Department of Natural Sciences, Mid Sweden University, 85170 Sundsvall, Sweden

**Keywords:** Al-ion battery, 99% Al 1% Fe alloy anode, cycling performance, corrosion, oxide film, Al_3_Fe particle

## Abstract

Considerable research has been devoted to the development of cathode materials for Al-ion batteries, but challenges remain regarding the behavior of aluminum anodes. Inert oxide (Al_2_O_3_) film on Al surfaces presents a barrier to electrochemical activity. The structure of the oxide film needs to be weakened to facilitate ion transfer during electrochemical activity. This study addresses oxide film challenges by studying Al alloy anodes with different iron content. The results reveal that using an anode of 99% Al 1% Fe in a cell increases the cycling lifetime by 48%, compared to a 99.99% Al anode. The improvement observed with the 99% Al 1% Fe anode is attributed to its fractional surface area corrosion being about 12% larger than that of a 99.99% Al anode. This is coupled to precipitation of a higher number of Al_3_Fe particles, which are evenly scattered in the Al matrix of 99% Al 1% Fe. These Al_3_Fe particles constitute weak spots in the oxide film for the electrolyte to attack, and access to fresh Al. The addition of iron to an Al anode thus offers a cheap and easy route for targeting the oxide passivating film challenge in Al-ion batteries.

## 1. Introduction

The rapid growth in electronic devices and grid storage applications has created a great need for low-cost and renewable energy storage systems. Currently the battery market is dominated by lithium-ion batteries (LIB), which have high power/energy density and a long cycle life [1,2,3,4,5,6,7,8,9]. However, sustainability concerns related to the scarcity of lithium, safety issues, and the high production costs for LIB are driving the development of alternative batteries [1,3,7,8]. Aluminum-based batteries are one of the candidates that could satisfy sustainability requirements. Al is abundant, and has a low cost, good safety, and good recyclability. In addition, the theoretical gravimetric capacity of Al (2980 mAh g^−1^) is significantly higher than that of metals such as Mg and Zn (2200 mAh g^−1^ and 820 mAh g^−1^, respectively). Only Li (3860 mAh g^−1^) has a higher gravimetric capacity. However, the theoretical volume capacity of Al (8040 mAh cm^−3^) is higher than that of Li (2060 mAh cm^−3^). These factors suggest Al as an attractive candidate for anode materials [2,3,4,7,9].

Various groups of materials have been studied as cathodes in Al-ion batteries using aqueous and non-aqueous systems. It reveals that an improvement in Al-batteries is still required from different aspects. For instance, in Al batteries using graphitic cathodes and non-aqueous electrolytes of [EMIMCl] and AlCl_3_, capacities are not still good enough [2]. In this system, Al species of AlCl_4_^−^ and Al_2_Cl_7_^−^ are coexisted and intercalated into graphitic layers [5,10]. While the majority of the research in Al-ion batteries to date has been devoted to the cathode material, far less work has focused on anode materials [9,11,12]. Nevertheless, to develop Al batteries resolving the problems that are attributed to the Al anode side are essential [9]. This urges us to explore the behavior of Al anodes in more detail in Al batteries during electrochemical activities. Al anodes present challenges involving self-corrosion, surface passivation, and the formation of dendrites. The native passivating oxide layer in Al surface metal is a serious obstacle in all Al-based batteries [9,13]. This inert oxide Al_2_O_3_ film disrupts ion/electron transformation during electrochemical operations and reduces cell efficiency [9,14,15,16,17,18]. One way to minimize the problem with the oxide layer is surface pretreatment of Al metal using procedures such as mechanical polishing, electrochemical polishing, and chemical etching [9,19,20]. However, Al metal is quickly re-passivated in contact with oxygen. It is important to note that the oxide film does not need to be entirely removed; the oxide structure need only be reconstructed to improve the ion/electron transfer for the charge/discharge process. One option for doing this involves adding alloying elements to the Al metal to produce an Al alloy. This strategy could create an oxide layer with more defect sites, providing more pathways for the electrolyte to reach the fresh Al surface and carry out electrostripping/deposition [9,21].

Iron is a main impurity element that is always found in Al metals. It has very low solubility (0.05 wt%) in Al and is precipitated as intermetallic phases, that is, as Al_3_Fe particles in the Al matrix. These phases exist even in Al with a purity marked as 99.99 [22,23,24,25,26,27]. These Al_3_Fe particles induce localized corrosion of the surrounding Al matrix due to galvanic coupling between the micro-particle, and the matrix [23,25]. In other words, Al_3_Fe phases in the Al matrix produce points where the passivating oxide layer is potentially weak when in contact with an electrolyte [23]. A previous paper [28] about an aqueous Al battery found that Al_3_Fe phases acted as beneficial sites for the electrolyte to eliminate the oxide barrier on an Al surface, resulting in enhanced electrochemical performance.

The purpose of this work is to study the performance of Al alloy anodes with different Fe content in Al-ion batteries, focusing on Al corrosion behavior in Al/graphitic cell using ionic liquid electrolyte of ([EMIMCl] and AlCl_3_). The correlation between cell performances and corrosion in Al anodes were explored in detail from a metallurgical perspective of the intermetallic phases. This would give an insight to approach the oxide film barrier of Al in Al-batteries.

## 2. Experimental

### 2.1. Preparation of Al Batteries

Al alloy foils with iron content of 0.01, 0.5, and 1 wt% (marked as 99.99% Al, 99.5% Al 0.5% Fe, and 99% Al 1% Fe, respectively) were used as the anode electrode. All Al foils were 100 µm thick and were purchased from Goodfellow, Friedberg, Germany. The cathode was a composite of nanographite (NG) and activated carbon with nanocellulouse binder, as described in [29,30]. The Al-NG batteries were assembled using 2025 coin-type cells with an ionic liquid electrolyte ([EMIMCl] and AlCl_3_ in 1:2 ratio; io-li-tec) in an argon-filled glove box. Whatman glass microfiber sheets (grade GF/D) were chosen as the separator. The assembled batteries were held for 4 h before starting the electrochemical tests. Additionally, symmetric cells of 99.99% Al, 99.5% Al 0.5% Fe, and 99% Al 1% Fe, respectively, were assembled using a similar coin-type cell, electrolyte and separator that were mentioned above.

### 2.2. Electrochemical Measurements

Cyclic voltammetry (CV) tests were carried out using a VersaSTAT 4 potentiostat, Sweden in a voltage range of 0.1 V to 3.0 V with scan rates of 5 mVs^−1^. Galvanostatic charge–discharge (GCD) measurements were conducted in a voltage range of 0.4 V to 2.2 V at a current density of 0.5 Ag^−1^ using a VersaSTAT 4 potentiostat. The capacities and current densities were computed based on the loading of the active material, which was about 4 mg/cm^2^. The electrochemical impedance spectroscopy measurements were performed with an amplitude of 10 mV in the frequency range of 100 kHz to 100 mHz for the as-assembled symmetric cells of 99.99% Al, 99.5% Al 0.5% Fe, and 99% Al 1% Fe, respectively.

### 2.3. Material Characterization

The surface morphology and microstructure of the Al alloy electrodes were investigated using a field emission scanning electron microscopy at 15 kV (MAIA3, TESCAN, Oxie, Sweden). Energy dispersive X-ray spectroscopy (EDX) (Oxford, UK) was also used to identify the chemical composition of the particles. 3D stereoscopic SEM images (Alicona, µeX, Sweden) were created to analyze and measure the corrosion volume in the Al electrodes. Structural characterization was also carried out using X-ray diffraction analysis (XRD, Bruker, Karlsruhe, Germany) with Cu-Kα radiation in the range of 2θ = 5–100° for Al alloy electrodes.

## 3. Results and Discussion

### 3.1. Electrochemical Analysis

**Figure 1:**
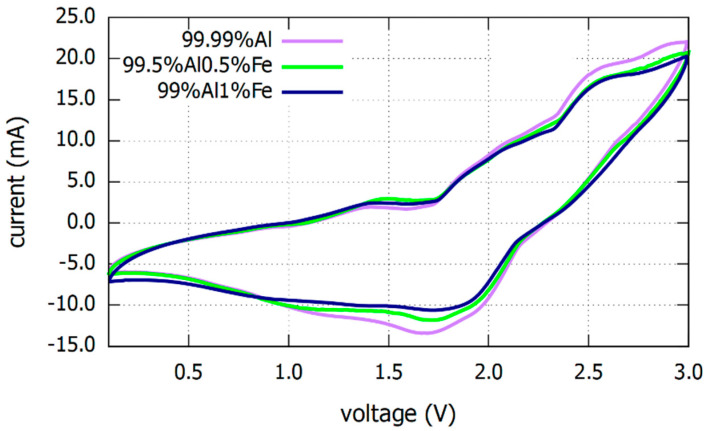
Cyclic voltammetry (CV) graphs of Al-NG battery using Al alloy anodes with various Fe content under the voltage range of 0.1–3V at a scan rate of 5 mVs^−1^ from cycle no 5.

The cyclic voltammetry (CV) graphs from Al-NG battery using Al alloy anode with different Fe content is illustrated in Figure 1. CV curves from all three Al-NG batteries showed a similar behavior. The cathodic and anodic peaks observed in CV graphs are probably related to AlCl_4_^−^ anions intercalation and de-intercalation in graphite layers when Al plating and stripping happens on the Al surface side. The peaks about 2.5 V are ascribed to the intercalation of AlCl_4_^–^ into the graphite layer, whereas the peaks about 1.7 V correspond to the de-intercalation of AlCl_4_^–^ from the graphite layers [12,31,32].

**Figure 2:**
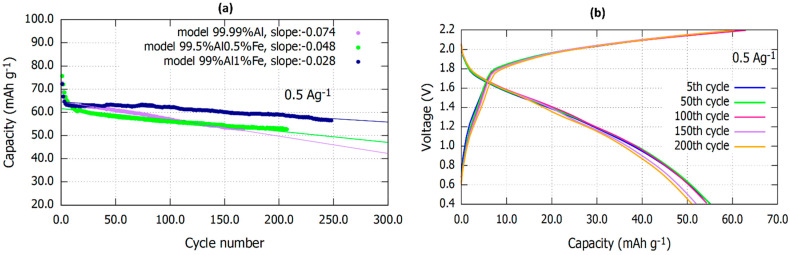
(**a**) Cycling performance of Al-NG battery using Al alloy anodes with various Fe content at a current density of 0.5 A g^−1^, (**b**) Galvanostatic charge/discharge curves of an Al-NG battery using 99% 1%Fe Al alloy anode at 5th, 50th, 100th, 150th, and 200th cycles for a current density of 0.5 A g^−1^.

Figure 2a shows the charge–discharge cycling performance of three different Al-NG cells using 99.99% Al, 99.5% Al 0.5% Fe, and 99% Al 1% Fe alloy anodes, respectively, in a voltage range of 0.4 to 2.2 V at current densities of 0.5 A g^−1^. As can be seen in Figure 2a, all three cells initially started with an approximately similar capacity, followed by a sharp drop after a few cycles (stabilizing period). After that, cells with 99.99% Al and 99.5 Al 0.5% Fe anodes revealed fast capacity degradation over cycling, while the cell with 99% Al 1% Fe showed less reduction in capacity. It should be mentioned that the cell with 99.99% Al anode revealed a capacity drop with linear behavior from the beginning, while the cell with 99.5 Al 0.5% Fe anodes exhibited a drop with a lower slope over cycling. It can also be stated that the cell with 99% Al 1% Fe started showing a drop in capacity approximately after 100 cycles, which became steeper over cycling. However, it can be seen from Table 1 that the cell with 99% Al 1% Fe anode showed a longer cycling life (248 cycles) compared to the 99.5% Al 0.5% Fe (207 cycles), and the 99.99% Al (167 cycles) anodes. It can be concluded that having some level of Fe content in the Al alloy anode, for #example, 1 wt%, enhanced the cycling behavior of Al-NG cells. It improved the capacity value and resulted in a longer cycling life. An example of galvanostatic charge/discharge curves for an Al-NG battery using a 99% 1%Fe Al alloy anode for various cycling numbers is illustrated in Figure 2b.

**Figure 3:**
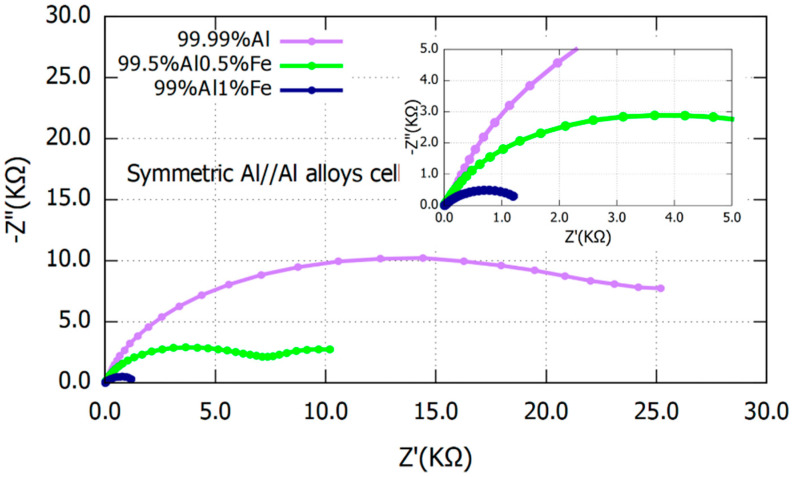
Electrochemical impedance spectra (EIS) for symmetrical cells of 99.99% Al, 99.5% Al 0.5% Fe, and 99% Al 1% Fe.

Electrochemical impedance spectroscopy measurements for symmetric cells that were constructed of 99.99% Al, 99.5% Al 0.5% Fe, and 99% Al 1% Fe, respectively, are revealed in Figure 3. The Nyquist plot exhibited a clearly larger diameter for 99.99% Al, compared to 99.5% Al 0.5% Fe and 99% Al 1% Fe anodes. This reveals a higher charge transfer resistance and thus higher corrosion resistance for 99.99% Al than two Al alloy anodes containing iron. It means that probably weaker native oxide films have been formed in 99.5% Al 0.5% Fe and 99% Al 1% Fe anodes from the beginning [14,33,34,35,36].

### 3.2. Material Analysis of Aluminum Electrodes

**Figure 4:**
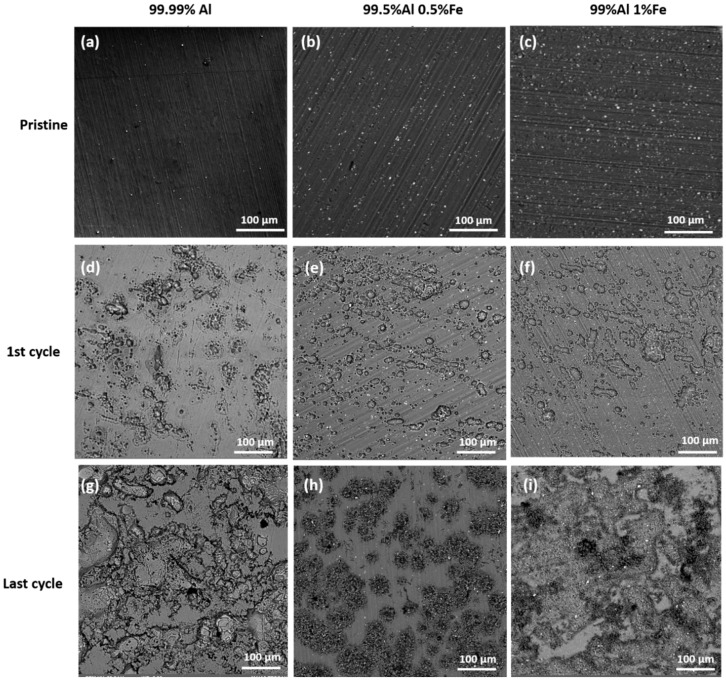
SEM backscattered images of pristine Al alloy surfaces: (**a**) 99.99% Al, (**b**) 99.5% Al 0.5% Fe, (**c**) 99% Al, 1% Fe. SEM backscattered images from Al alloy anodes surfaces after first charge–discharge cycles: (**d**) 99.99% Al, (**e**) 99.5% Al 0.5% Fe, (**f**) 99% Al 1% Fe. SEM images of the Al alloy anode surfaces from last charge–discharge cycles: (**g**) 99.99% Al, (**h**) 99.5% Al 0.5% Fe, (**i**) 99% Al 1% Fe.

Figure 4 shows images of the Al alloy anodes’ surfaces with various Fe contents, as pristine, after the first cycle and after the last cycle, respectively. The SEM images of pristine Al anodes in Figure 4a,b, and c reveal a number of particles (white dots) scattered in the Al matrix. The population density of these particles in the Al matrix increases with increasing Fe contents; Table 1. Figure 4c shows that the particles are evenly distributed over the matrix. The particle sizes vary between 1 and 10 µm, but they are mainly smaller than 5 µm (see the supplementary images in Figure S1). EDX analysis of the particles shows that they correspond to Al_3_Fe intermetallic phases (Figure S2). Additionally, the existence of Al_3_Fe phases has been confirmed using the XRD technique (shown in Figure S3). As stated earlier, Fe is the main impurity found in Al, and has a very low solubility of about 0.05 wt% in Al. This leads to the precipitation of Fe-containing intermetallic phases such as Al_3_Fe in bulk aluminum [20,21,25,37]. Thus, it is expected that more Al_3_Fe phases will be observed as the solubility limits are exceeded, as is the case for 99.5% Al 0.5% Fe and 99% Al 1% Fe in Figure 4b,c, respectively. It should be noted that most research in Al-ion batteries has used a very pure Al (Al ≥ 99.9). The manufacturing cost of high-purity Al (99.9% purity) is substantially higher than that of commercial pure Al (99.5% purity) [24,38]. Thus, using high-purity Al (99.9% purity) introduces another challenge from a commercialization perspective when developing Al-ion batteries. In addition, the results in Figure 2 show that commercial pure Al (99.5% purity) gives a better result than high-purity Al (Al ≥ 99.9). Moreover, while in practice, Al_3_Fe phases exist in a matrix of a very pure Al (Al ≥ 99.9), its role has not been noticed and discussed in Al anode performance.

Figure 4d–f are SEM images for the 99.99% Al, 99.5% Al 0.5% Fe, and 99% Al 1% Fe anodes, respectively, after the first cycle. High numbers of small and large corrosion sites are visible on both the 99.5% Al 0.5% Fe and 99% Al 1% Fe surfaces, while fewer corrosion sites appear on the 99.99% Al surface. Many of the corrosion sites on both 99.5% Al 0.5% Fe and 99% Al 1% Fe have formed either around or adjacent to the Al_3_Fe phases (white particles), as shown in Figure S4. As stated above, this local corrosion is a consequence of galvanic coupling between Al_3_Fe particles and the Al matrix [23,25].

The SEM images of 99.99% Al, 99.5% Al 0.5% Fe, and 99% Al 1% Fe electrodes taken from the last cycles are shown in Figure 4g,h,i, respectively. Figure 4i shows that the corrosion area has grown significantly over the whole surface and is interconnected in the 99% Al 1% Fe anode. Only a small fraction of the area has remained unaffected. By contrast, there are larger unaffected surface areas on both the 99.99% Al and 99.5% Al 0.5% Fe electrodes, with many corrosion spots only partly interconnected, and in some places appearing as isolated islands in Figure 4g,h.

**Figure 5:**
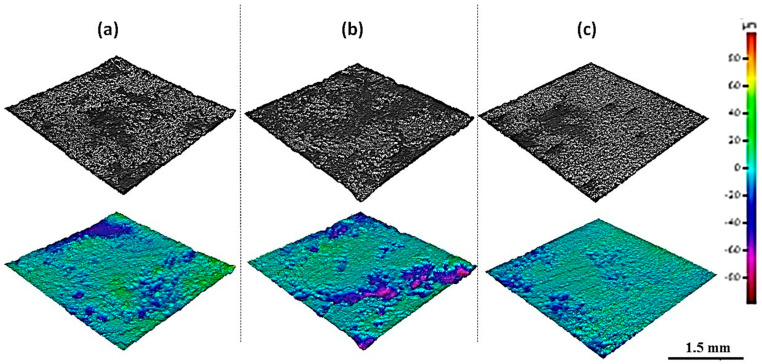
Three−dimensional SEM image of Al alloy electrode surfaces from the last cycles visualizing corrosion morphology: (**a**) 99.99% Al, (**b**) 99.5% Al 0.5% Fe, (**c**) 99% Al 1% Fe (scale for color contrast is in µm).

**Figure 6:**
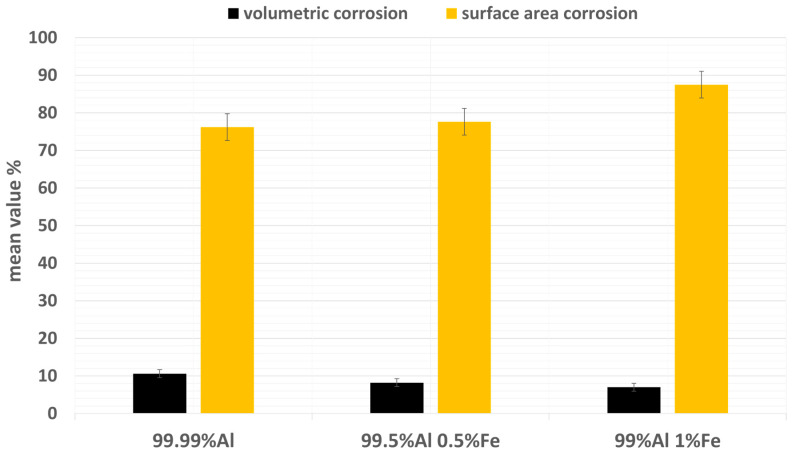
Volumetric and surface area corrosion for Al electrodes with different Fe contents.

A 3D stereoscopic SEM imaging technique was applied to the images of the Al alloy anode electrodes after the last cycle to visualize the corrosion morphology, and to quantify the corrosion (Figure 5). A smooth corrosion morphology is seen on almost the entire surface of 99% Al 1% Fe (Figure 5c), whereas Figure 5a,b for the other anodes clearly show some areas with a rough corrosion morphology. A higher number of deep corrosion pits, ranging in depth from 20 to 60 µm, are agglomerated in 99.99% Al and 99.5% Al 0.5% Fe electrodes, compared to the 99% Al 1% Fe anode. The volumetric and surface area corrosion for each Al electrode was calculated using the 3D SEM images and illustrated in Figure 6. The volumetric corrosion measurement is based on the sum of the volumes of corrosion pits over the whole Al surface. The surface area corrosion corresponds to the sum of the corroded surface area across the whole Al surface. The volumetric and surface area corrosion obtained for 99.99% Al, 99.5% Al 0.5% Fe, and 99% Al 1% Fe electrodes are given in Table 1. The volumetric corrosion of all Al electrodes is very similar. However, the 99.99% Al electrode with very low Fe content had relatively larger volumetric corrosion than the other Al electrodes. By contrast, there were noticeable differences in surface area corrosion. The largest surface area corrosion occurred in the 99% Al 1% Fe electrode (87.5%), and the smallest surface area corrosion was observed in the 99.99% Al electrode (76.2%). It can be concluded that the addition of Fe content to 1 wt% in Al results in larger surface-area corrosion. This is also seen in Figure 4i (99%Al 1% Fe electrode), which shows that by the last cycle, a major part of the surface had been corroded. Conversely, the volumetric corrosion from the two Al electrodes with lower Fe content up to 0.5 wt% shows slightly larger values compared to the 1 wt% Fe electrode. The average corrosion depth was calculated (Table 1) and estimated that the depth of corrosion pits has been reduced by 50% in the 99% Al 1% Fe anode, compared with corrosion pits formed in 99.99% Al anode. This can also be observed in the 3D SEM image (Figure 5), where deeper corrosion pits (20–60 µm) were formed in electrodes with lower Fe content up to 0.5 wt%, compared to the 1 wt% Fe electrode.

Al-NG cells using Al alloy anodes with higher Fe contents, 99% Al 1% Fe and 99.5% Al 0.5% Fe, respectively, have about 48% and 24% longer cycling lifetime, compared with 99% Al 1% Fe (as seen in Table 1). The corresponding Al electrodes 99.99% Al, 99.5% Al 0.5% Fe, and 99% Al 1% Fe exhibit fractional surface area corrosion of 76.2%, 77.6%, and 87.5%, respectively. This finding implies that a correlation between larger surface area corrosion and enhanced electrochemical performance exists. From a corrosion point of view, it can be concluded that the electrochemical performance of an Al-ion battery correlates with surface area corrosion and not volumetric corrosion. The results can also be interpreted as indicating that once corrosion pits reach a certain depth, for example, 20 µm (dark blue and pink area in Figure 5), they no longer favor electrochemical activity. Reference [23] also states that the accumulation of corrosion products on pitting reduces electrochemical activity. This phenomenon would probably be more significant in deeper corrosion pits, as was observed in the 99.99% Al and 99.5% Al 0.5% Fe anodes.

References [23,28] state that Al_3_Fe phases are the preferred sites for the electrolyte to break the oxide barrier and initiate corrosion. A higher number of Al_3_Fe particles provides a larger number of possible locations to break through, and thus a larger surface area for the electrolyte to be involved in electrochemical activity. The calculations of particle densities seen in Table 1 show that the addition of 1 wt% Fe in Al led to the precipitation of 4(10^7^) Al_3_Fe particles per cm^2^, which is 4 times and 50 times larger than the number of Al_3_Fe particles precipitated in Al alloy containing 0.5 and 0.01 wt% Fe, respectively. That is why a larger surface area has been corroded in the 99% Al 1% Fe electrode. Here, it can be concluded that about a 12% increase in surface area corrosion occurred in the 99% Al 1% Fe anode, compared to 99.99% Al, which was due to an increase in population densities of Al_3_Fe particles. This calculation provides an estimate of the contribution of the population density of Al_3_Fe particles to Al surface corrosion, and thus to electrochemical performance. In other words, the cell performance enhancement observed with the 99% Al 1% Fe anode is coupled to the higher number of Al_3_Fe particles precipitated in 99% Al 1% Fe.

Very pure and expensive Al (99.99%) still contains Al_3_Fe as impurities, and variations in the population of such phases can cause random variations in battery performance. This is also could be seen in Figure S5 that the reproducibility of capacity profiles from Al-NG batteries using the Al 99.99% anode indicates a level of randomness, compared with cells using 99.5% Al 0.5% Fe and 99% Al 1% Fe anode alloys, Figures S6 and S7, respectively. In addition, there are always defects in the oxide layer that are not easy to control. It means that Al anodes do not always have a homogenous microstructure, and, thus, the oxide barrier may behave differently from sample to sample. These results presented in this study suggest that adding a certain level of Fe, namely, 1 wt%, produces a more homogenous microstructure in an Al alloy electrode due to the even distribution of Al_3_Fe phases, as is shown in Figure 4c. It is a strategy to evenly weaken the oxide barrier. This, in turn, optimizes Al anode behavior during electrochemical performance in an Al-ion battery.

## 4. Conclusions

The behavior of aluminum alloy anodes with different iron content was studied for aluminum-ion batteries. Adding 1 wt% Fe to the Al alloy electrode enhanced the cell performance and resulted in an Al-ion battery with a longer lifetime and a higher capacity. The enhancement in the electrochemical performance of Al with 1 wt% Fe was attributed to the higher surface area corrosion of the Al alloy anode. It was concluded that the addition of Fe up to 1 wt% facilitates the fragmentation of the oxide barrier on the Al surface and, thus, electrolyte access to bulk Al. This mechanism occurs through the precipitation of a high number of Al_3_Fe intermetallic phases that are evenly distributed in the Al matrix. This research thus suggests that a simple metallurgical treatment could improve Al alloy anode behavior in Al-ion batteries.

## Figures and Tables

**Table 1:** Summary of data from cycling performance, SEM analysis, and corrosion calculations.

Al Alloy Electrode	Lifetime (Cycle #)	Al_3_Fe Particle (#/cm^2^)	Surface Area Corrosion (%)	Volume Corrosion (%)	Average Corrosion Depth (µm)	Capacity Degradation Slope	Initial Capacity Cycle#5 (mAh g^−1^)	Final Capacity (mAh g^−1^)
99.99% Al	167	~8 (10^5^)	76.2	10.6	~10	0.074	64.1	52.8
99.5% Al 0.5% Fe	207	~10^7^	77.6	8.2	~8	0.048	65.3	52.7
99% Al 1% Fe	248	~4 (10^7^)	87.5	7	~5	0.028	63.3	56.6

Number (#).

## Supplementary Materials

The following supporting information can be downloaded at: https://www.mdpi.com/article/10.3390/ma16030933/s1. Experimental details; EDX analysis of intermetallic particles; cyclic voltammetry graphs. References [39,40] are cited in the supplementary materials.

## References

[1] Yang H., Li H., Li J., Sun Z., He K., Cheng H.M., Li F. (2019). The rechargeable aluminum battery: Opportunities and challenges. Angew. Chem. Int. Ed..

[2] Ru Y., Zheng S., Xue H., Pang H. (2019). Different positive electrode materials in organic and aqueous systems for aluminium ion batteries. J. Mater. Chem. A.

[3] Leisegang T., Meutzner F., Zschornak M., Münchgesang W., Schmid R., Nestler T., Meyer D.C. (2019). The aluminum-ion battery: A sustainable and seminal concept?. Front. Chem..

[4] Faegh E., Ng B., Hayman D., Mustain W.E. (2021). Practical assessment of the performance of aluminium battery technologies. Nature Energy.

[5] Tu J., Song W.L., Lei H., Yu Z., Chen L.L., Wang M., Jiao S. (2021). Nonaqueous rechargeable aluminum batteries: Progresses, challenges, and perspectives. Chem. Rev..

[6] Ejigu A., Le Fevre L.W., Elgendy A., Spencer B.F., Bawn C., Dryfe R.A. (2022). Optimization of Electrolytes for High-Performance Aqueous Aluminum-Ion Batteries. ACS Appl. Mater. Interfaces.

[7] Yuan D., Zhao J., Manalastas Jr W., Kumar S., Srinivasan M. (2020). Emerging rechargeable aqueous aluminum ion battery: Status, challenges, and outlooks. Nano Mater. Sci..

[8] Mckerracher R.D., Holland A., Cruden A., Wills R.G.A. (2019). Comparison of carbon materials as cathodes for the aluminium-ion battery. Carbon.

[9] Jiang M., Fu C., Meng P., Ren J., Wang J., Bu J., Sun B. (2022). Challenges and Strategies of Low-Cost Aluminum Anodes for High-Performance Al-Based Batteries. Adv. Mater..

[10] Kim J., Raj M.R., Lee G. (2021). High-Defect-Density Graphite for Superior-Performance Aluminum-Ion Batteries with Ultra-Fast Charging and Stable Long Life. Nano-Micro Lett..

[11] Yang H., Wu F., Bai Y., Wu C. (2020). Toward better electrode/electrolyte interfaces in the ionic-liquid-based rechargeable aluminum batteries. J. Energy Chem..

[12] Long Y., Li H., Ye M., Chen Z., Wang Z., Tao Y., Yang Q.H. (2021). Suppressing Al dendrite growth towards a long-life Al-metal battery. Energy Storage Mater..

[13] Guo M., Fu C., Jiang M., Bai Y., Zhang J., Sun B. (2020). High performance aluminum foam-graphite dual-ion batteries and failure analysis. J. Alloys Compd..

[14] Dong T., Ng K.L., Wang Y., Voznyy O., Azimi G. (2021). Solid electrolyte interphase engineering for aqueous aluminum metal batteries: A critical evaluation. Adv. Energy Mater..

[15] Zhao Q., Zachman M.J., Al Sadat W.I., Zheng J., Kourkoutis L.F., Archer L. (2018). Solid electrolyte interphases for high-energy aqueous aluminum electrochemical cells. Sci. Adv..

[16] Wu F., Zhu N., Bai Y., Gao Y., Wu C. (2018). An interface-reconstruction effect for rechargeable aluminum battery in ionic liquid electrolyte to enhance cycling performances. Green Energy Environ..

[17] Wang H., Gu S., Bai Y., Chen S., Wu F., Wu C. (2016). High-voltage and noncorrosive ionic liquid electrolyte used in rechargeable aluminum battery. ACS Appl. Mater. Interfaces.

[18] Ambroz F., Macdonald T.J., Nann T. (2017). Trends in aluminium-based intercalation batteries. Adv. Energy Mater..

[19] Choi S., Go H., Lee G., Tak Y. (2017). Electrochemical properties of an aluminum anode in an ionic liquid electrolyte for rechargeable aluminum-ion batteries. Phys. Chem. Chem. Phys..

[20] Go H., Michael R.R., Tak Y., Lee G. (2022). Electrochemically surface-modified aluminum electrode enabling high performance and ultra-long cycling life Al-ion batteries. Electroanalysis.

[21] Chen H., Xu H., Zheng B., Wang S., Huang T., Guo F., Gao C. (2017). Oxide film efficiently suppresses dendrite growth in aluminum-ion battery. ACS Appl. Mater. Interfaces.

[22] Davis J.R. (1993). Aluminum and Aluminum Alloys.

[23] Vargel C. (2020). Corrosion of Aluminium.

[24] Ambat R., Davenport A.J., Scamans G.M., Afseth A. (2006). Effect of iron-containing intermetallic particles on the corrosion behaviour of aluminium. Corros. Sci..

[25] Jin Z., Cai C., Hashimoto T., Yuan Y., Kang D., Hunter J., Zhou X. (2021). The behaviour of iron-containing intermetallic particles in aluminium alloys in alkaline solution. Corros. Sci..

[26] Seri O. (2006). Surface Treatment for Corrosion Resistant Aluminium Alloys by Removing Intermetallic Phases. Mater. Sci. Forum.

[27] Li J., Dang J. (2017). A summary of corrosion properties of Al-rich solid solution and secondary phase particles in Al alloys. Metals.

[28] Rastabi S.A., Razaz G., Hummelgård M., Carlberg T., Blomquist N., Örtegren J., Olin H. (2022). Metallurgical investigation of aluminum anode behavior in water-in-salt electrolyte for aqueous aluminum batteries. J. Power Sources.

[29] Blomquist N., Engström A.C., Hummelgård M., Andres B., Forsberg S., Olin H. (2016). Large-scale production of nanographite by tube-shear exfoliation in water. PLoS ONE..

[30] Blomquist N., Koppolu R., Dahlström C., Toivakka M., Olin H. (2020). Influence of substrate in roll-to-roll coated nanographite electrodes for metal-free supercapacitors. Sci. Rep..

[31] Wang D.Y., Wei C.Y., Lin M.C., Pan C.J., Chou H.L., Chen H.A., Dai H. (2017). Advanced rechargeable aluminium ion battery with a high-quality natural graphite cathode. Nat. Commun..

[32] Liu S., Yang J., Song X., Wang Y., Zhang W., Chhowalla M. (2022). Microwave-Reduced Graphene Oxide for Aluminum Batteries. ACS Appl. Nano Mater..

[33] Sanad M.M., Azab A.A., Taha T. (2022). A Introduced oxygen vacancies in cadmium ferrite anode materials via Zn2+ incorporation for high performance lithium-ion batteries. Mater. Sci. Semicond. Process..

[34] Nandi S., Das S.K. (2019). Realizing a low-cost and sustainable rechargeable aqueous aluminum-metal battery with exfoliated graphite cathode. ACS Sustain. Chem. Eng..

[35] Lim J., Jeong G., Seo K., Lim J., Park S., Ju W., Sim U. (2022). Controlled optimization of Mg and Zn in Al alloys for improved corrosion resistance via uniform corrosion. Mater. Adv..

[36] Ran Q., Shi H., Meng H., Zeng S.P., Wan W.B., Zhang W., Jiang Q. (2022). Aluminum-copper alloy anode materials for high-energy aqueous aluminum batteries. Nat. Commun..

[37] Razaz G., Carlberg T. (2019). On the Dissolution Process of Manganese and Iron in Molten Aluminum. Metall. Mater. Trans. A.

[38] Cho Y.J., Park I.J., Lee H.J., Kim J.G. (2015). Aluminum anode for aluminum–air battery–Part I: Influence of aluminum purity. J. Power Sources.

[39] Basariya M.R., Roy R.K., Pramanick A.K., Srivastava V.C., Mukhopadhyay N.K. (2015). Structural transition and softening in Al–Fe intermetallic compounds induced by high energy ball milling. Mater. Sci. Eng. A.

[40] Bendjeddou L., Debili M.Y., Fekrache A., Boulkhessaim S. (2009). Structure and phase transformation in HF melted Al–Fe–Ti alloys. Phys. Procedia.

