# Supplementary material for: Aluminum Alloy Anode with Various Iron Content Influencing the Performance of Aluminum-Ion Batteries
Source: Materials (Basel). 2023 Jan 18;16(3):933. doi: 10.3390/ma16030933 (PMC9917774; doi:10.3390/ma16030933)
Supplement: Supplementary file 1 [file materials-16-00933-s001.zip › materials-2150122-supplementary.pdf]

Supporting Information

Aluminum Alloy Anode with Various Iron Content Influencing the Performance of Aluminum-Ion Batteries

Ghadir Razaz *, Shahrzad Arshadirastabi, Nicklas Blomquist, Jonas Örtegren, Torbjörn Carlberg, Magnus Hummelgård and Håkan Olin

Department of Natural Sciences, Mid Sweden University, 85170 Sundsvall, Sweden
* Correspondence: ghadir.razaz@miun.se

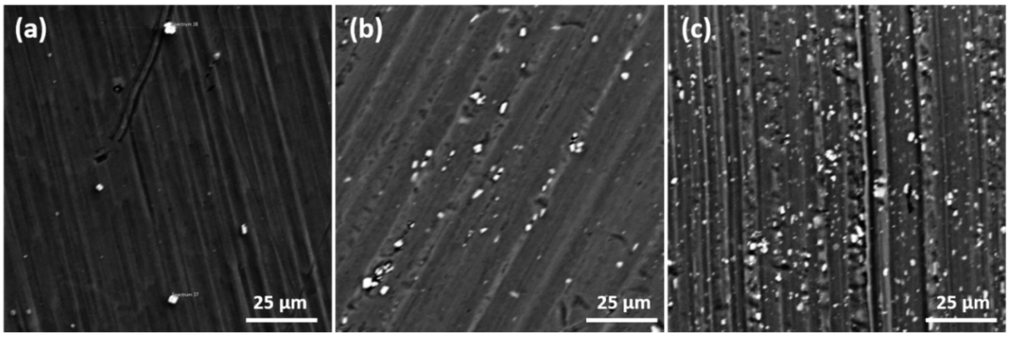

Figure S1. SEM backscattered images of Pristine Al surfaces shown in Fig 2 from a higher magnification revealing particles: (a) 99.99% Al, (b) 99.5% Al 0.5%Fe, (c) 99% Al 1% Fe.

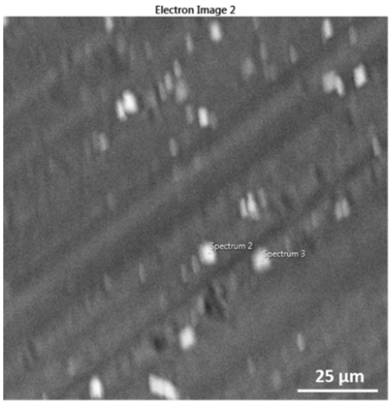

Spectrum 2

Element	Weight %	σ
Al	72.3	4.5
Fe	27.7	4.5

Spectrum 3

Element	Weight %	σ
Al	76.0	5.2
Fe	24.0	5.2

Figure S2. EDX analysis of particles precipitated in pristine Al matrix.

As could be seen in Fig.S3, the peak intensities corresponding to Al_3Fe phases are significantly smaller compared to the Al matrix (α-Al). This is due to the very small amount of such phases compared to the Al matrix [1,2].

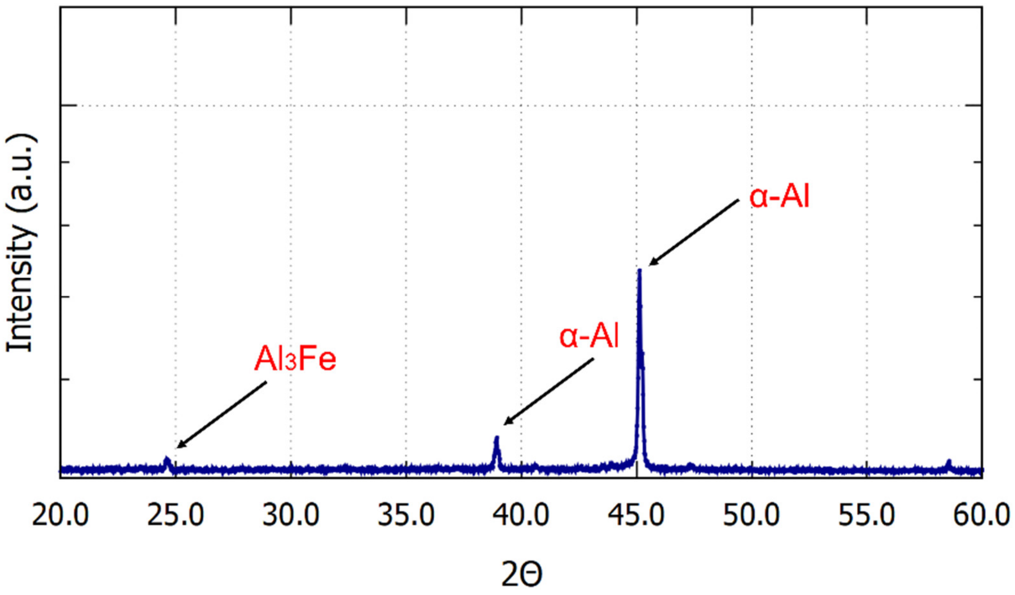

Figure S3. Spectrum from X-ray diffraction performed on pristine 99%Al 1%Fe alloy.

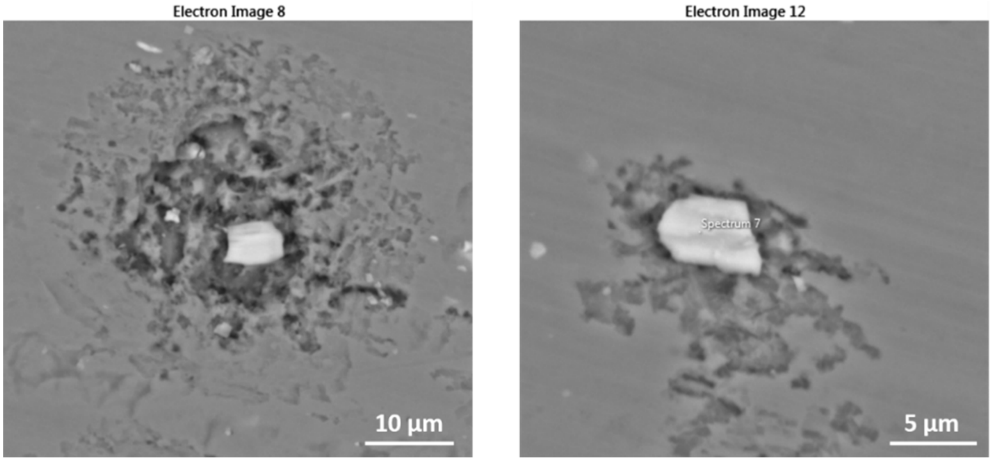

Figure S4. SEM images of corrosion sites in Al matrix around Al_3Fe particles in different locations after 1 st charge–discharge cycle.

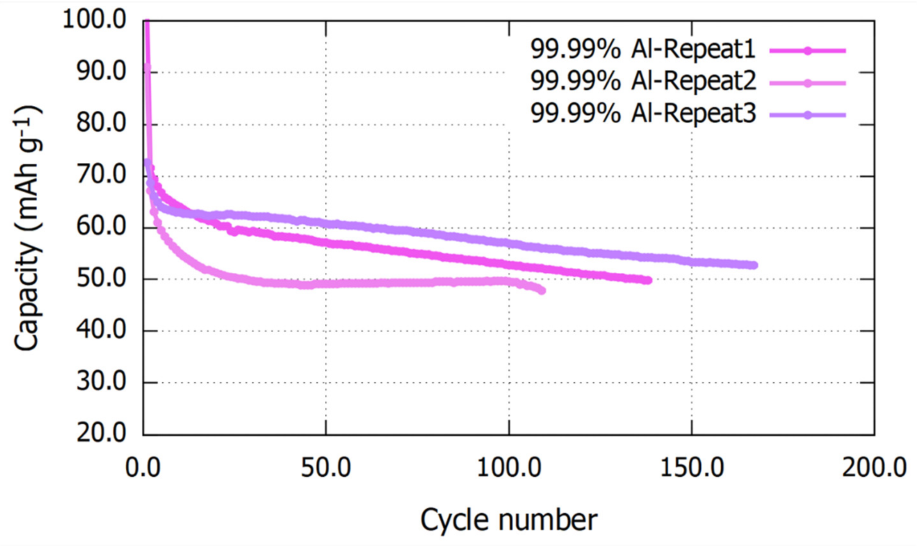

Figure S5. Cycling performance of Al-NG battery using 99.99% Al anode at a current density of 0.5 A g^{-1} showing reproducibility of capacity profiles.

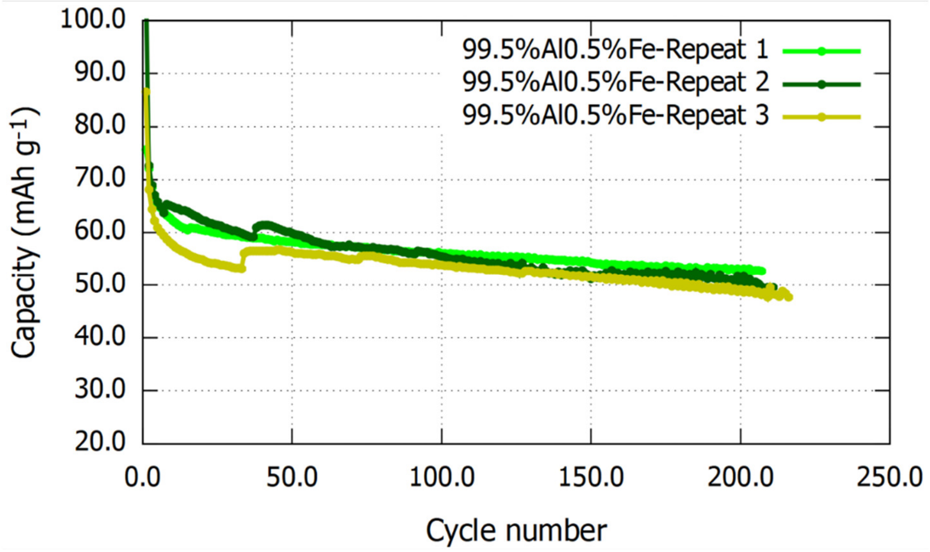

Figure S6. Cycling performance of Al-NG battery using 99.5% Al alloy anode at a current density of 0.5 A g^{-1} showing reproducibility of capacity profiles.

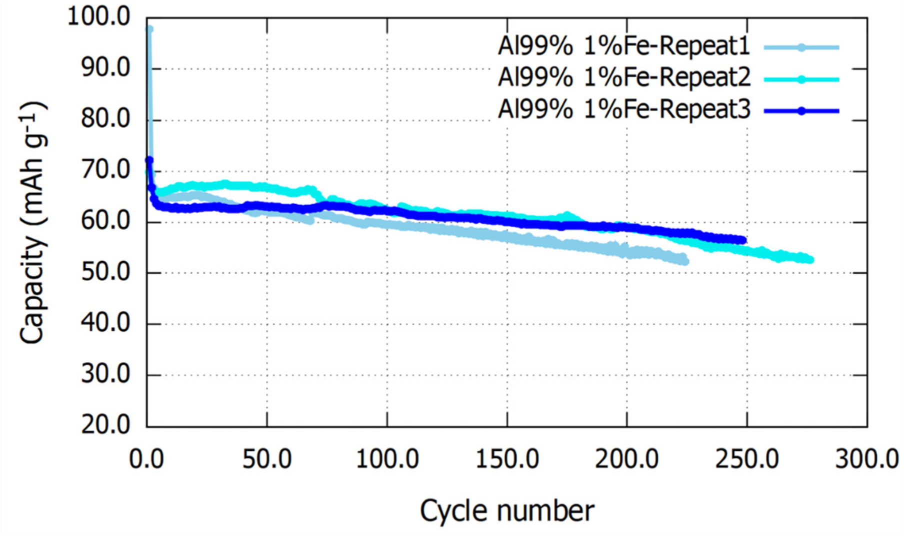

Figure S7. Cycling performance of Al-NG battery using 99% 1%Fe Al alloy anode at a current density of 0.5 A g^{-1} showing reproducibility of capacity profiles.

References

[1] Basariya, M. R., Roy, R. K., Pramanick, A. K., Srivastava, V. C., & Mukhopadhyay, N. K. (2015). Structural transition and softening in Al–Fe intermetallic compounds induced by high energy ball milling. *Materials Science and Engineering: A*, *638*, 282-288.

[2] Bendjeddou, L., Debili, M. Y., Fekrache, A., & Boulkhessaim, S. (2009). Structure and phase transformation in HF melted Al–Fe–Ti alloys. *Physics procedia*, *2*(3), 1113-1118.
